# High-performance glass filters for capturing and culturing circulating tumor cells and cancer-associated fibroblasts

**DOI:** 10.1038/s41598-023-31265-9

**Published:** 2023-03-13

**Authors:** Hiromasa Tanaka, Daijiro Iwata, Yuki Shibata, Tetsunari Hase, Daisuke Onoshima, Naoyuki Yogo, Hirofumi Shibata, Mitsuo Sato, Kenji Ishikawa, Ikuo Nagasawa, Yoshinori Hasegawa, Makoto Ishii, Yoshinobu Baba, Masaru Hori

**Affiliations:** 1 Center for Low-Temperature Plasma Sciences, Nagoya University, Furo-cho, Chikusa-ku, Nagoya, 464-8601 Japan; 2 Innovative Technology Laboratories, AGC Inc., 1-1 Suehirocho Tsurumi-ku, Yokohama, 230-0045 Japan; 3 Department of Respiratory Medicine, Nagoya University Graduate School of Medicine, 65 Tsurumai-cho, Showa-ku, Nagoya, 466-8550 Japan; 4 Institute of Nano-Life-Systems, Institute of Innovation for Future Society, Nagoya University, Nagoya, 464-8601 Japan; 5 Division of Host Defense Sciences, Department of Integrated Health Sciences, Nagoya University Graduate School of Medicine, Daikominami 1-1-20, Higashi-ku, Nagoya, Japan; 6 National Hospital Organization, Nagoya Medical Center, 4-1-1, Sannomaru, Naka-ku, Nagoya, 460-0001 Japan

**Keywords:** Lab-on-a-chip, Nanobiotechnology, Diagnostic markers

## Abstract

Various liquid biopsy methods have been developed for the non-invasive and early detection of diseases. In particular, the detection of circulating tumor cells (CTCs) and cancer-associated fibroblasts (CAFs) in blood has been receiving a great deal of attention. We have been developing systems and materials to facilitate such liquid biopsies. In this study, we further developed glass filters (with various patterns of holes, pitches, and non-adhesive coating) that can capture CTCs, but not white blood cells. We optimized the glass filters to capture CTCs, and demonstrated that they could be used to detect CTCs from lung cancer patients. We also used the optimized glass filters for detecting CAFs. Additionally, we further developed a system for visualizing the captured cells on the glass filters. Finally, we demonstrated that we could directly culture the captured cells on the glass filters. Based on these results, our high-performance glass filters appear to be useful for capturing and culturing CTCs and CAFs for further examinations.

## Introduction

Early diagnosis is important for the early treatment of diseases, and greatly impacts the longevity and health of individuals and society as a whole^1–5^. Exosomes and cell-free DNA/RNAs are promising biomarkers for liquid biopsy since they provide important information about diseases, such as cancer^6–9^. Circulating tumor cells (CTCs) are tumor cells that circulate in the blood, and the detection of CTCs has attracted much attention, because they provide direct information about cancer, and may be used as markers to predict disease progression even in early-stage cancer patients^10–14^. CTCs are also important for elucidating the mechanisms of cancer metastasis^15^. However, the number of CTCs in the blood of cancer patients is extremely low, so improvements in the CTC capture efficiency and the ability to culture the isolated CTCs are needed for further genetic and molecular analyses. In recent years, single-cell analysis methods have been extensively developed^16–18^, but it remains difficult to analyze a single CTC. Thus, a breakthrough is needed for capturing and analyzing CTCs from liquid biopsies. Cancer-associated fibroblasts (CAFs), which circulate in blood, are also important cells that are analyzed in liquid biopsies^19–22^ since they can provide information about cancer progression that is different from the information obtained from CTCs. CAFs can negatively or positively regulate cancer progression, e.g., CAFs that express Meflin inhibit pancreatic carcinogenesis^23^.

Various nanobiodevices have been developed for the early diagnosis of cancer^24–28^. We have previously developed low-autofluorescence fluoropolymer membrane filters for capturing CTCs^29^. We used ethylene tetrafluoroethylene for the filters, and we were able to successfully capture CTCs without capturing white blood cells using these filters. We have also further developed glass filters with micro-sized holes^30^. Since glass is often used for culturing cells, we expected that it would be possible to culture CTCs on the glass filters; however, such glass filters remained to be developed at the time.

In this study, we developed glass filters to capture CTCs and CAFs. Several patterns of glass filters with varying hole sizes were tested, and we found that some patterns could capture CTCs while allowing white blood cells to pass through. CTCs from lung cancer patients were captured using the glass filters. In addition, CAFs were also efficiently captured using them. Furthermore, we developed a system that we called Filtering and Real-time Observation Glass (FROG)-CHIP for visualizing how CTCs and CAFs are captured on the glass filters. Finally, we demonstrated that the captured CTCs and CAFs could be cultured directly on the glass filters. These results suggest that they may be a promising tool for the early diagnosis of cancer.

## Results and discussion

### Preparation of the CTC isolation system with a glass filter

**Figure 1:**
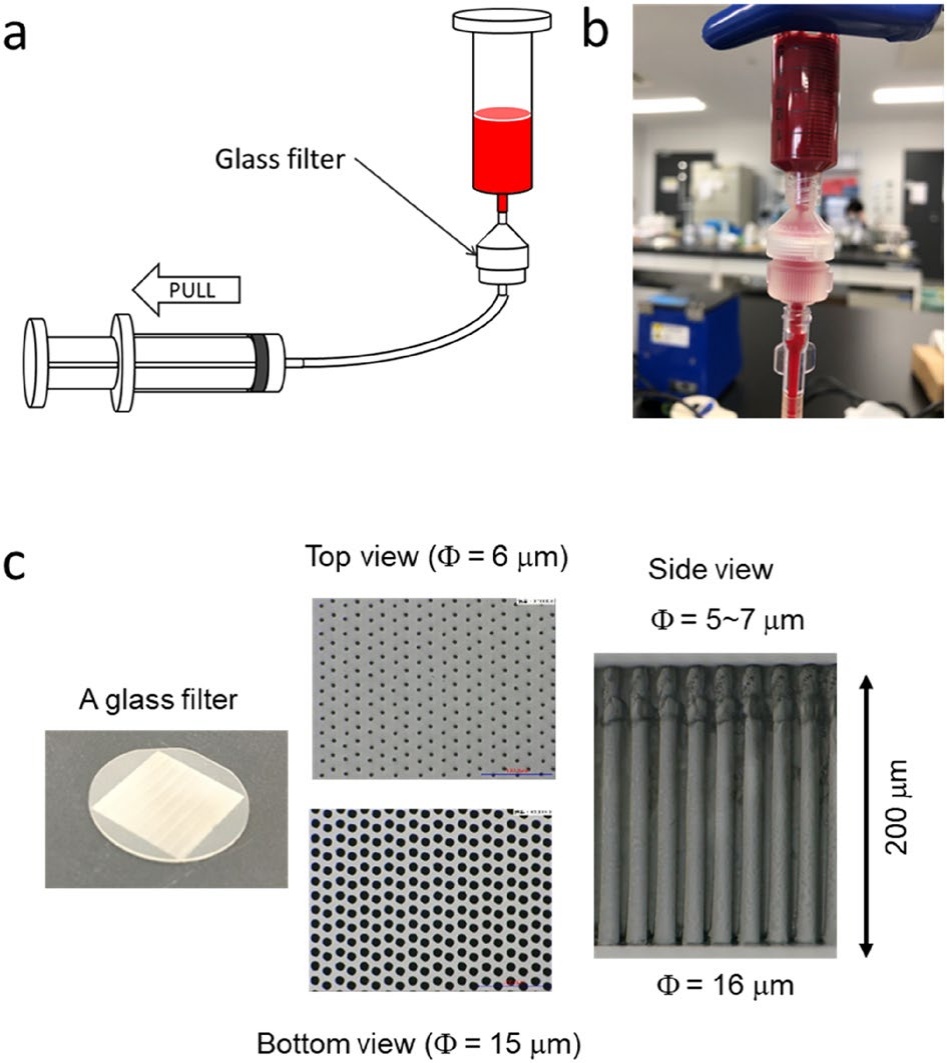
The experimental setup to capture CTCs and CAFs. (**a**) Schematic of the experimental setup to capture CTCs and CAFs. (**b**) Photograph of the experimental setup around the glass filter. (**c**) Top view, bottom view, and side view of the glass filter.

We constructed a CTC isolation system with a glass filter as previously described (Fig. 1a,b)^29^. We created glass filters with hundreds of thousands of holes and a 200-μm thickness (Fig. 1c). The 200-μm thickness of the slides was chosen as it was determined to ensure sufficient strength as a glass filter, and that it would be easy to observe under a microscope. We designed glass filters with several patterns of hole diameter sizes (large hole size/small hole size = 15 μm/6 μm, 17 μm/8 μm, or 19 μm/10 μm), and assessed their cell-capturing efficiency as well as how many white blood cells would be eliminated.

### The relationship between the flow rate and differential pressure

**Figure 2:**
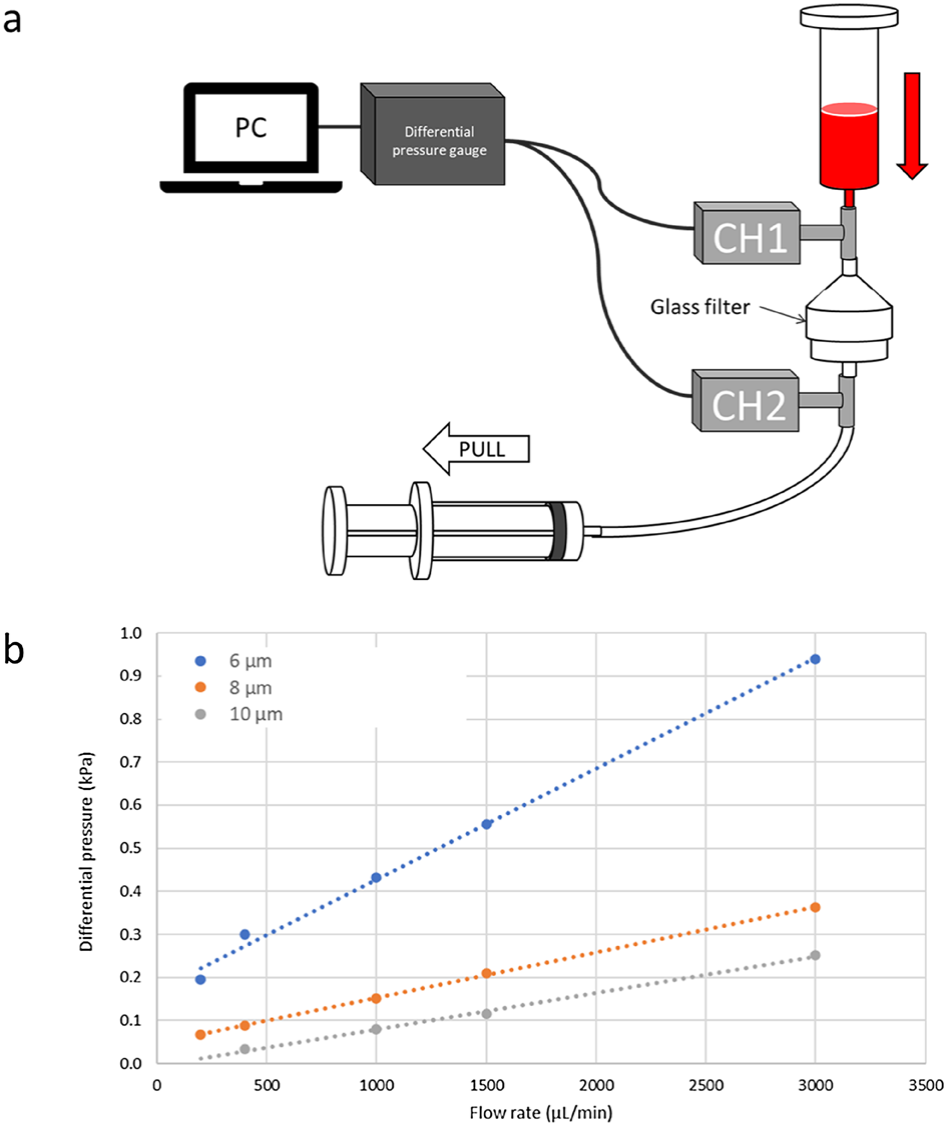
Measurements of the differential pressure of glass filters for capturing CTCs and CAFs. (**a**) Schematic of the experimental setup for measuring the differential pressure of the glass filters. (**b**) The relationship between the flow rate and differential pressure of the glass filters with holes (hole diameter of 6, 8, or 10 μm).

To investigate the relationship between the flow rate and differential pressure, we measured the differential pressure between the areas immediately before and after the glass filter (Fig. 2). The blood samples were prepared by mixing equal volumes of blood and phosphate-buffered saline (PBS). Then, the blood samples were drawn using a syringe pump (Fig. 2a). Figure 2b shows the relationship between the flow rate and differential pressure in glass filters of several patterns. The differential pressure was proportional to the flow rate, and the slope depended on the hole size of the glass filter. The slope was larger for the glass filters with a large hole size/small hole size of 15 μm/6 μm than for those with the sizes of 17 μm/8 μm or 19 μm/10 μm.

### Capturing CTCs from blood using various glass filters

**Figure 3:**
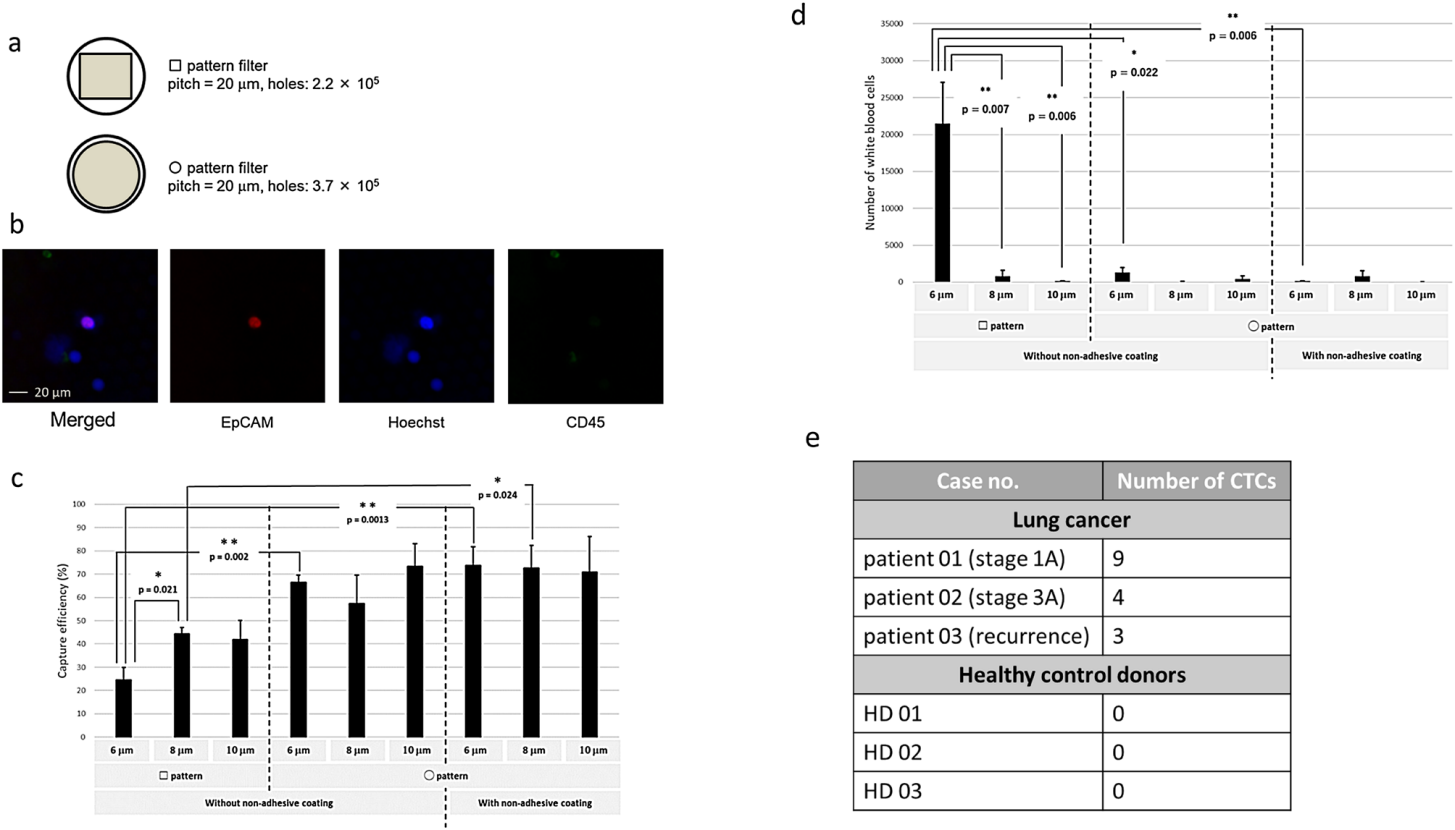
CTC capture test using the glass filters. (**a**) Schematic of the various glass filters. (**b**) CTCs captured on a glass filter. (**c**) The CTC capture efficiency of various glass filters. (**d**) Cell counts of white blood cells on the various glass filters. (**e**) Numbers of captured CTCs on glass filters from the blood of lung cancer patients and healthy control donors.

To determine the optimal glass filter, we performed spike tests using cultured cells H358 as model cells of CTCs with various types of glass filters (Fig. 3). We initially used glass filters with a square pattern, but then we also developed glass filters with a circular pattern (Fig. 3a) since more holes could be fit into the filter with such a pattern (3.7 × 10^5^ holes) when compared to a square pattern (2.2 × 10^5^ holes). Many holes are needed for removing red blood cells and white blood cells and stabilizing the differential pressure. Figure 3b shows a typical image of CTC (patient cells) captured on a glass filter. Epithelial cellular adhesion molecule (EpCAM)-positive, Hoechst-positive, and CD45-negative cells were taken to be CTCs (patient cells), and we calculated the CTC capture efficiency.

With the glass filters with a square pattern, the CTC (H358) capture efficiency increased as the small hole size became larger (Fig. 3c); when the small hole sizes were 6 μm, 8 μm, and 10 μm, the capture efficiencies were approximately 24%, 44%, and 42%, respectively. When we used the glass filters with a circular pattern, the capture efficiency significantly improved; when the small hole size was 6 μm, the capture efficiency was approximately 66%. Figure 3d shows the counts of CD45-positive and Hoechst-positive cells, which represent white blood cells. When the small hole size of the glass filters with a square pattern was 6 μm, many white blood cells remained on the glass filters. However, when the small hole size of the glass filters with a circular pattern was 8 μm, a smaller number of white blood cells remained on the glass filters. We believe the reason many more white blood cells remained on the glass filters with a square pattern than on those with a circular pattern is that a higher density of holes in the circular pattern than in the square pattern. In addition, we believe the reason the capture efficiency was better with the circular pattern than with the square pattern is that the differential pressure is larger with the square pattern than with the circular pattern. To further decrease the number of white blood cells on the glass filters, we developed glass filters with a circular pattern and non-adhesive coating; when the small hole size was 8 μm, the capture efficiency was approximately 73%, and few white blood cells remained on the glass filters.

Finally, we performed CTC isolation from the blood samples of lung cancer patients and healthy donors using the glass filters with a circular pattern and non-adhesive coating (small hole size = 8 μm). We successfully isolated 3 to 9 CTCs from the blood of each lung cancer patients, and no CTCs were isolated from the blood of the healthy donors (Fig. 3e).

### Capturing CAFs from blood using glass filters

**Figure 4:**
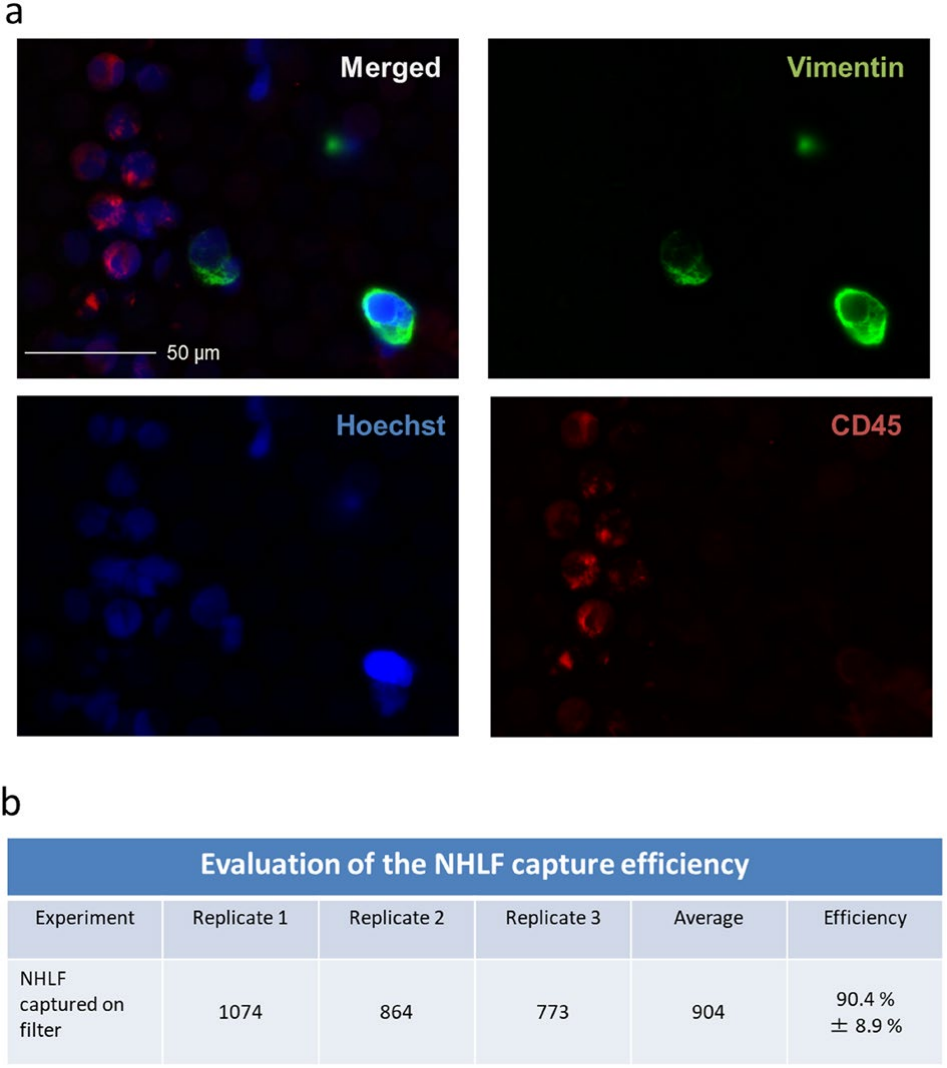
CAF capture test using glass filters. (**a**) CAFs captured on a glass filter. (**b**) Capture efficiency of CAF.

To investigate whether CAFs can also be captured on the glass filters with a circular pattern and non-adhesive coating (small hole size = 8 μm), we performed spike tests using normal human lung fibroblast (NHLF) cc-2512 cells as model cells of CAFs (Fig. 4). We fixed the blood samples with 0.4% paraformaldehyde so that soft cells such as fibroblasts can be easily captured on the glass filter. The captured cells were stained with anti-Vimentin antibody to identify CAFs (cc-2512 cells), and with Hoechst and anti-CD45 antibody to identify white blood cells (Fig. 4a). The capture efficiency was 90.4% ± 8.9%. However, CTCs that have undergone epithelial to mesenchymal transition (EMT) are positive for Vimentin, so the numbers of (EMT-independent) CTCs and CAFs remain to be re-evaluated by using other CAF markers such as FAP in the future^31^. These results suggest that our glass filters can capture not only (EMT-independent) CTCs, but also CAFs. Thus, these glass filters appear to be useful as a tool for the early diagnosis of cancer since much information about cancer can be gained from the captured CTCs and CAFs.

### Visualizing cells captured on glass filters

**Figure 5:**
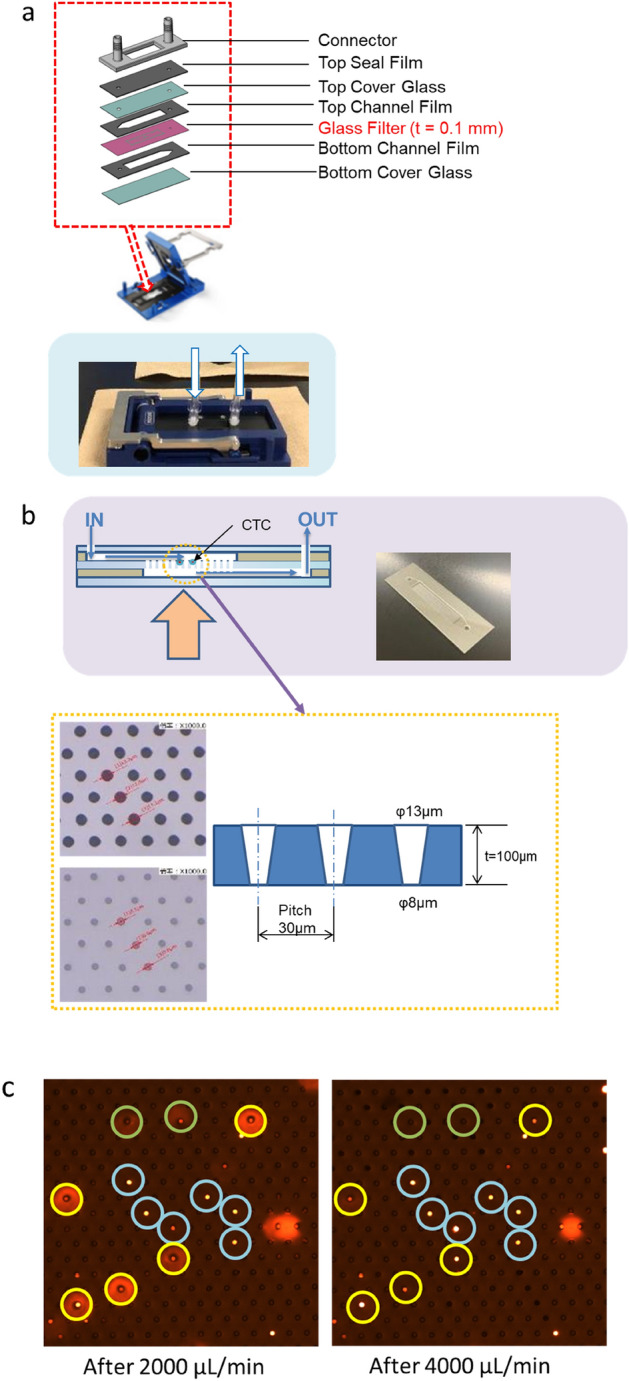
Observation of CTCs and CAFs using the FROG-CHIP system. (**a**) A schematic of the FROG-CHIP device. (**b**) A schematic of the glass filter in the FROG-CHIP device. (**c**) Images of cells observed on the FROG-CHIP system.

When capturing cells from blood with a size filter, it is important to determine the conditions, such as the optimal flow rate, pressure, and pore size, that will enable leukocytes to pass through the pores, but not the cells of interest. However, since the size and hardness of cells differ depending on the type of cells, many tests need to be performed to determine the optimal conditions, which takes time. Therefore, to make it easier to understand the state of cells during filtering so that the optimal conditions can be determined, we devised and manufactured a channel device that can be used to directly observe the state of cell capture (Fig. 5), i.e., the FROG-CHIP system.

For the proof of concept of the FROG-CHIP system, we spiked the system with 3000 H358 cells in PBS, and observed the cells as the flow rate was changed from 1000 μL/min to 10,000 μL/min. After reaching a flow rate of 2000 μL/min, cells began to be captured on the glass filter (Fig. 5c). After reaching a flow rate of 4000 μL/min, some cells started to pass through the glass filter, while other cells remained on the filter.

Next, blood samples were prepared by mixing equal volumes of blood and PBS, and cc2512 cells were spiked into the blood samples. The blood samples were injected into the FROG-CHIP system, and the flowing cells were monitored using a microscope (Supplemental Data 1). We successfully observed how the cc2512 cells were captured on the glass filters, and how the white blood cells passed through the glass filters.

These results suggest that the FROG-CHIP system is a powerful tool for understanding how some cells are captured, and how some cells pass through filters.

### Culturing of the captured CTCs on the glass filters

**Figure 6:**
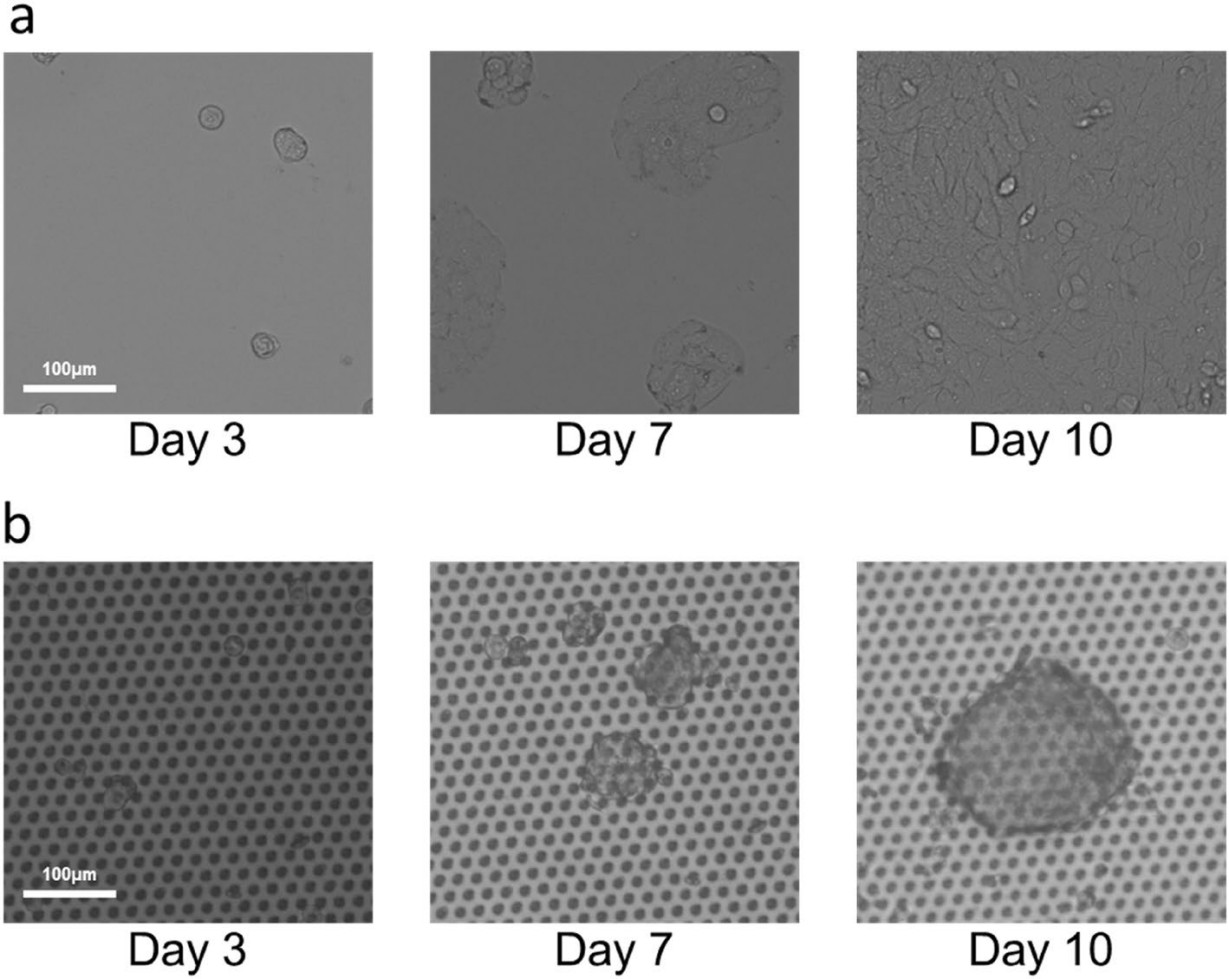
Culturing of the captured CTCs on the glass filter. H358 cells after isolation on a petri dish (**a**) and a glass filter with non-adhesive coating (**b**).

Since the density of CTCs and CAFs in blood is generally low, especially in early-stage cancer patients, it is often difficult to secure the number of cells (clones) required for further analyses, such as genetic analysis. In addition, it is hard to culture cells from only a few cells. To investigate whether it would be possible to culture cells directly on our glass filters (glass filters with a circular pattern and non-adhesive coating (large hole size/small hole size = 15 μm/6 μm)), we cultured the captured H358 cells on the glass filters using RPMI-medium (Fig. 6). As a positive control, we cultured H358 cells on a commercialized petri dish (Fig. 6a). The cells spread and grew on the petri dish. In contrast, the H358 cells that were captured on the glass filters formed spheroids as they grew on the glass filters with non-adhesive coating (Fig. 6b) and without non-adhesive coating (Supplemental Fig. 1). Live/dead staining revealed that the spheroids (H358 cells) cultured on the glass filters with and without non-adhesive coating were viable 10 days after culturing the cells (Supplemental Fig. 2). These results suggest that our glass filters are suitable for the culturing of CTCs for further analyses. Immunomagnetic capture methods such as Cell Search is widely used, however, it is limited to recover only EpCAM positive CTCs^32^. In addition to the Cell Search, various technologies to isolate CTCs have been developed^31^. EMT-independent detection methods of CTCs have been developed^33^, and immunomagnetic negative enrichment methods have been also developed^34^. We have previously developed a size based capture method using membrane filters with low-autofluorescence (ETFE film filters)^29^, and in this study, we have developed the glass filters so that CTCs can be cultured on the glass filters (Supplemental Table 1). However, culturing established cell line models differs greatly from culturing primary CTCs from patients, so further studies are needed to establish culturing primary CTCs from patients.

## Conclusions

We have developed a CTC and CAF isolation system using glass filters. We demonstrated that the glass filters could capture CTCs and CAFs in spike tests, and we also captured CTCs from the blood of lung cancer patients. In addition, we constructed a system for visualizing how the CTCs and CAFs are captured on the glass filters. Finally, we successfully cultured the captured cells directly on the glass filters. These results suggest that our glass filters may be useful as a tool for the early diagnosis of cancer. Further studies are needed to demonstrate sufficient sensitivity and specificity for the future clinical applications.

## Materials and methods

### Constructing the CTC- and CAF-capturing system

We developed a system for capturing CTCs and CAFs on glass filters as previously described (Fig. 1a,b)^29^. A glass filter was set in a filter holder (SWINNEX SX0001300), and an input syringe (TERUMO ss-10LZ) was set on the upper side of the filter holder. The blood samples were prepared by mixing 3.75 mL of fresh blood with an EDTA anti-coagulants and 3.75 mL of PBS. In the spike test, 1000 cells were spiked into the blood sample. For CAFs, 0.4% paraformaldehyde in PBS was added into the blood sample to fix the cells. The blood sample was added into the input syringe, then drawn into a waste syringe (TERUMO SS-20LZ) using a syringe pump (KD Scientific Legato200). The blood sample was passed through the filter at a flow rate of 1 mL/min for 7.5 min. Then, PBS was added into the input syringe, and passed through the filter at a flow rate of 1 mL/min for 5 min to wash the glass filter. For CAFs, 240 μL of blocking buffer (5% normal goat serum and 0.3% Triton X-100) was passed through the filter at a flow rate of 4 μL/min for 60 min. Then, 200 μL of a cell staining solution, which contained 1 μg/mL Hoechst 33342 (Dojindo Wako Pure Chemical Industries) and anti-CD45 antibody (Alexa Fluor® 647, 1:20 dilution; BioLegend), was passed through the filter at a flow rate of 4 μL/min for 50 min. The cell staining solution also contained a cocktail of phycoerythrin-labeled anti-EpCAM antibodies (1:3 dilution; BD Biosciences) to identify CTCs, or fluorescein-5-isothiocyanate-labeled anti-Vimentin antibodies (1:20; Abcam) to identify CAFs. Then, PBST (PBS containing 0.1% Tween 20) was added into the input syringe, and passed through the filter at a flow rate of 150 μL/min for 20 min to wash the glass filter. Captured cells were observed using a fluorescence microscope (Keyence BZ-X710). Total 6 × 8 images taken with 4× field of view were combined so that the entire field of view of the glass filter was covered. Then we counted all the cells captured on the glass filter to calculate the capture efficiency of the CTCs/CAFs.

### Preparation of glass filters

We created an optically transparent glass membrane filter with hundreds of thousands of bored-through and precisely arranged cross-sectionally tapered holes using laser and wet etching. The diameters of the larger and smaller holes (big hole size/small hole size) in the glass filters were 15 μm/6 μm, 17 μm/8 μm, or 19 μm/10 μm, and the thickness of the glass filters was fixed at 200 μm (Fig. 1c).

### Cell lines and cultures

The lung cancer cell line H358 was obtained from the American Type Culture Collection (Manassas, VA), and was grown in RPMI-1640 (Sigma-Aldrich, St. Louis, MO) supplemented with 10% fetal bovine serum and penicillin (100 U/mL)-streptomycin (100 μg/mL) under an atmosphere of 5% CO_2_ at 37 °C. The normal human lung fibroblast cell line cc-2512 was obtained from Lonza, and was grown in Fibroblast Growth Medium-2 BullertKit™.

### Clinical samples

5 mL of fresh blood samples with an EDTA anti-coagulants obtained from patients with pathologically diagnosed lung cancer at Nagoya University Hospital, Japan, were analyzed in this study. The time delay between blood collection from patients and processing the blood with the capture device were 2–6 h. The Ethics Review Committee of Nagoya University Graduate School of Medicine approved this study (No. 2017-0034), and written informed consent was obtained from the patients before blood collection. Commercially available ethylenediaminetetraacetic acid-treated tubes were used for whole blood collection. All methods were performed in accordance with the relevant guidelines and regulations.

### Measurement of the differential pressure

The differential pressure between the areas immediately before and after the glass filters were measured using blood pressure monitoring kits (DX300, Nihon Kohden), bridge amplifiers (FE221, AD Instruments), and an analog-to-digital converter (ML826 PowerLab 2/26, AD Instruments; Fig. 2a).

### The filtering and real-time observation glass (FROG)–CHIP system

A flow path was created with a resin film above and below the glass filter, and the top and bottom were sandwiched between cover glasses (Fig. 5a). The filter was made of glass with a thickness of 0.1 mm, and the hole size was 13 μm on the front side and 8 μm on the back side. For the resin sheet in the flow path, silicon rubber with a thickness of 500 μm was used on the input side, and polyethylene terephthalate film with a thickness of 100 μm was used on the output side. A 0.2-mm thick cover glass was used. The connector was made of polypropylene, and was manufactured using a 3D printer. Each part was coated with a non-cell-adhesive coating. The upper cover glass was machined with two 1-mm diameter holes for blood input and output, and the inputs and outputs were connected via a connector (Fig. 5b). Each component and connector of the flow path device was crimped using Micronit’s Fluidic Connect PRO, and after the test, it could be disassembled.

### Statistical analysis

All data are presented as the mean ± standard deviation. Statistical analysis of differences between groups was performed using the Student’s *t*-test. A P-value < 0.05 was considered to indicate a statistically significant difference.

## Supplementary Information

Supplementary Information.

## Data Availability

The data that support the findings of this study are available from the corresponding author upon reasonable request.
