# Supplementary material for: High-performance glass filters for capturing and culturing circulating tumor cells and cancer-associated fibroblasts
Source: Sci Rep. 2023 Mar 13;13:4130. doi: 10.1038/s41598-023-31265-9 (PMC10011408; doi:10.1038/s41598-023-31265-9)
Supplement: Supplementary file 1 — Supplementary Information. [file 41598_2023_31265_MOESM1_ESM.docx]

**High-performance glass filters for capturing and culturing circulating tumor cells and cancer-associated fibroblasts**

**Hiromasa Tanaka^1, *^, Daijiro Iwata^2^, Yuki Shibata^1^, Tetsunari Hase^3^, Daisuke Onoshima^4^, Naoyuki Yogo^3^, Hirofumi Shibata^3^, Mitsuo Sato^5^, Kenji Ishikawa^1^, Ikuo Nagasawa^2^, Yoshinori Hasegawa^3,6^, Makoto Ishii^3^, Yoshinobu Baba^4^, and Masaru Hori^1^**

^1^ Center for Low-temperature Plasma Sciences, Nagoya University, Furo-cho, Chikusa-ku, Nagoya 464-8601, Japan

^2^ Innovative Technology Laboratories, AGC Inc. 1-1 Suehirocho Tsurumi-ku Yokohama 230-0045, Japan

^3^ Department of Respiratory Medicine, Nagoya University Graduate School of Medicine, 65 Tsurumai-cho, Showa-ku, Nagoya 466-8550, Japan

^4^ Institute of Nano-Life-Systems, Institute of Innovation for Future Society, Nagoya University, Nagoya 464-8601, Japan

^5^ Division of Host Defense Sciences, Department of Integrated Health Sciences, Nagoya University Graduate School of Medicine, Daikominami 1-1-20, Higashi-ku, Nagoya, Japan

^6^ National Hospital Organization, Nagoya Medical Center, 4-1-1, Sannomaru, Naka-ku Nagoya 460-0001, Japan

*To whom correspondence should be addressed:

E-mail: [htanaka@plasma.engg.nagoya-u.ac.jp](mailto:htanaka@plasma.engg.nagoya-u.ac.jp)

**Supplemental Data**

**a**

<https://nuss.nagoya-u.ac.jp/s/5fMbJ9BoTw6ATMe>

**b**

<https://nuss.nagoya-u.ac.jp/s/tfgnWMbQ4ooTwHC>

**Supplemental Data 1:** **Movies in the FROG-CHIP experiments.** (a) NHLF (cc-2512) cells on the FROG-Chip device (b) Blood cells (most of them are white blood cells) on the FROG-Chip device.

**
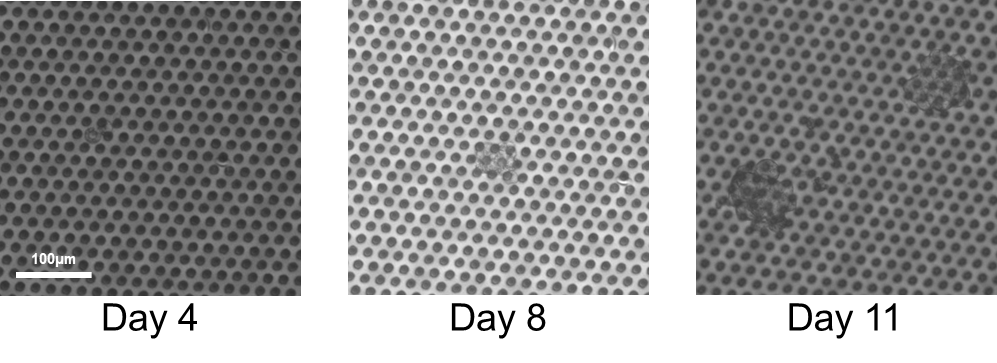
**

**Supplemental Figure 1: Culturing of the captured CTCs on the glass filter.** H358 cells after isolation on a glass filter without non-adhesive coating.

**
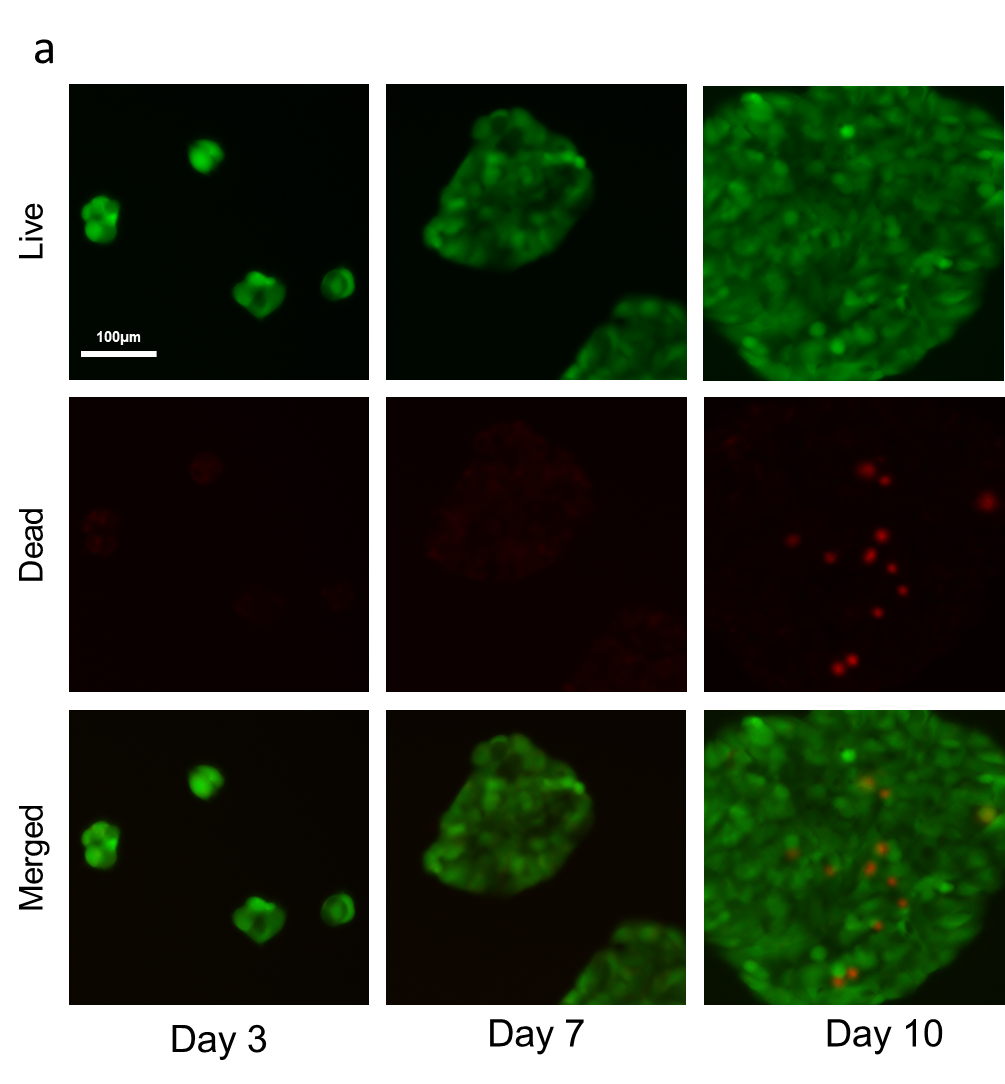
**

**
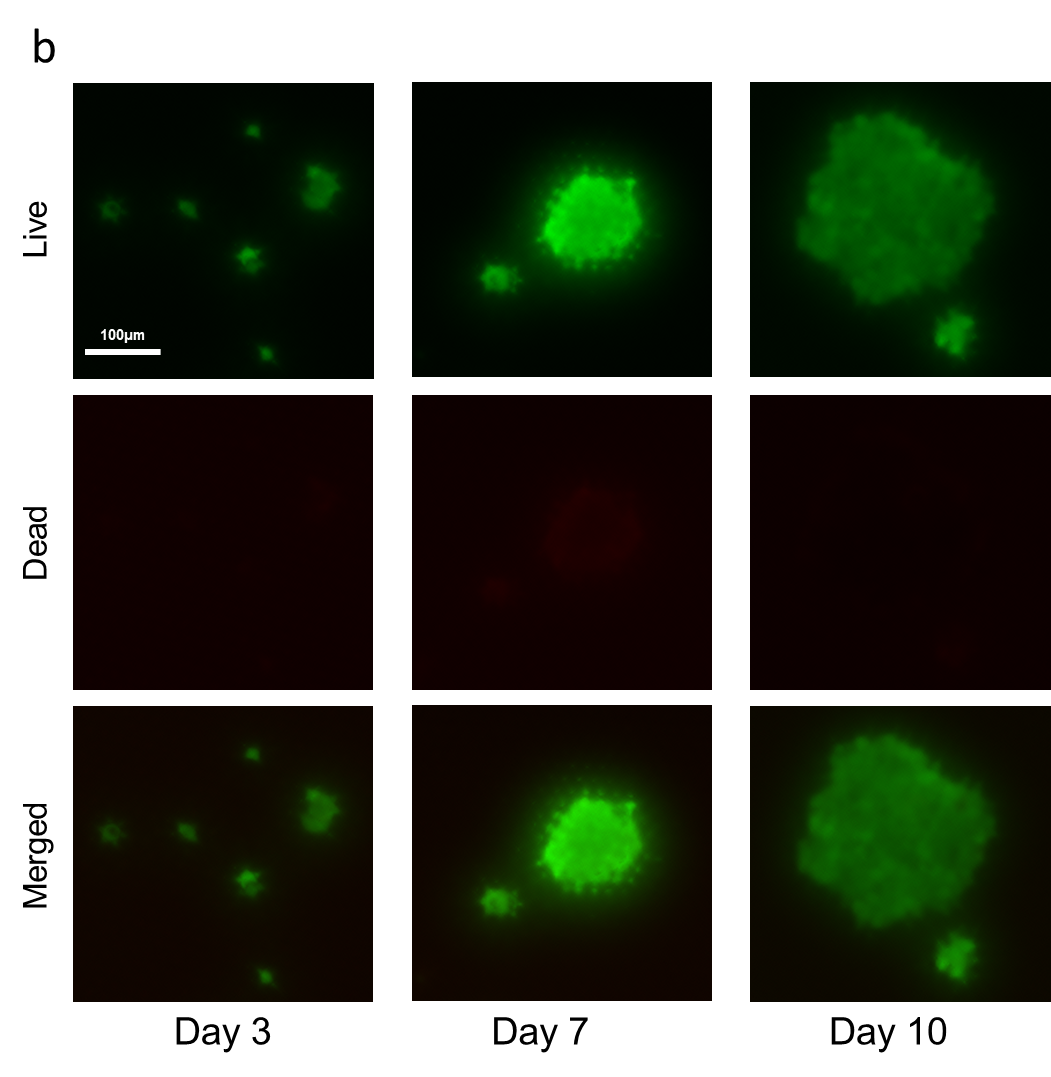
**

**
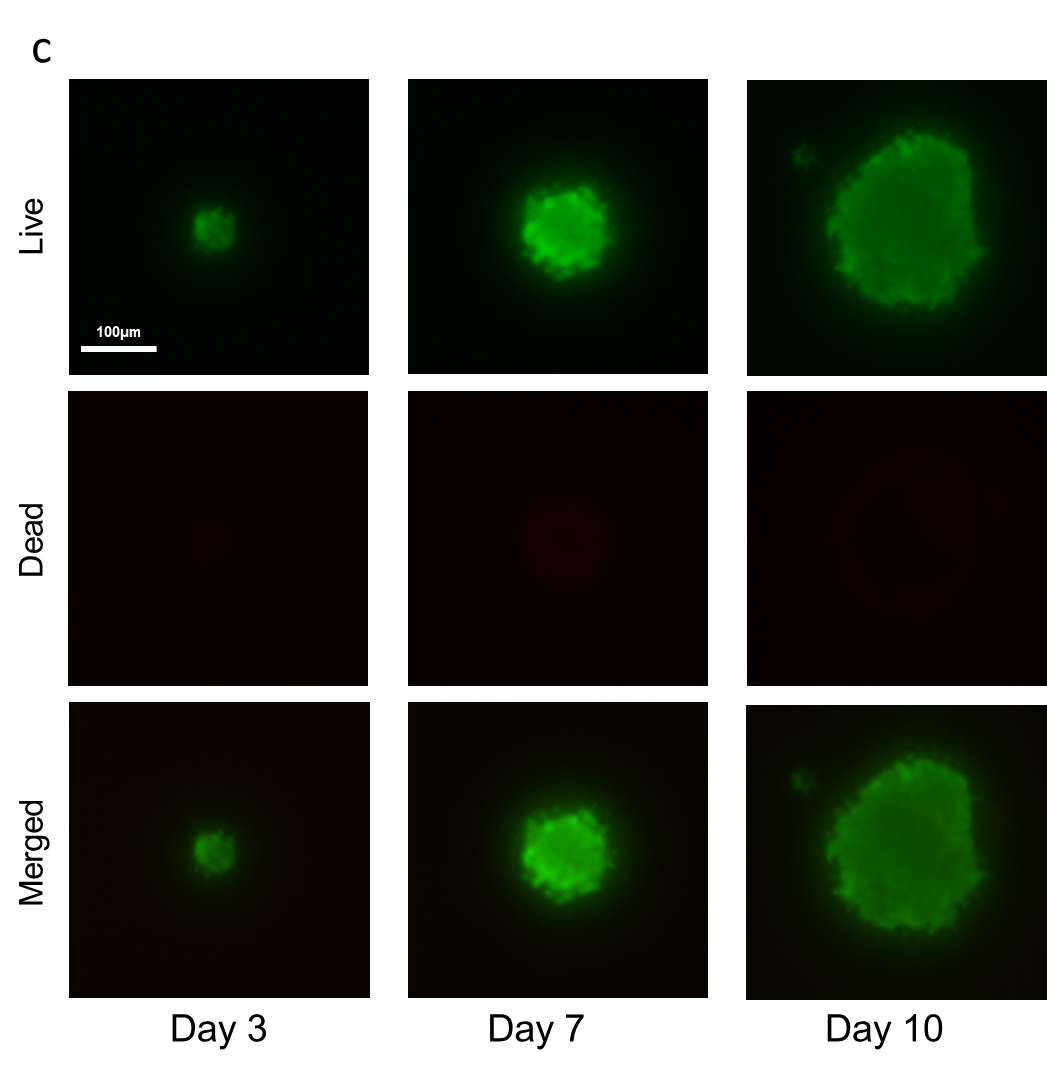
**

**Supplemental Figure 2: Live/dead judgement of cultured CTCs on the glass filter.** Live cells (green) and dead cells (red) cultured for 3, 7, and 10 days on a petri dish (a), a glass filter with non-adhesive coating (b), and a glass filter without non-adhesive coating (c).

**
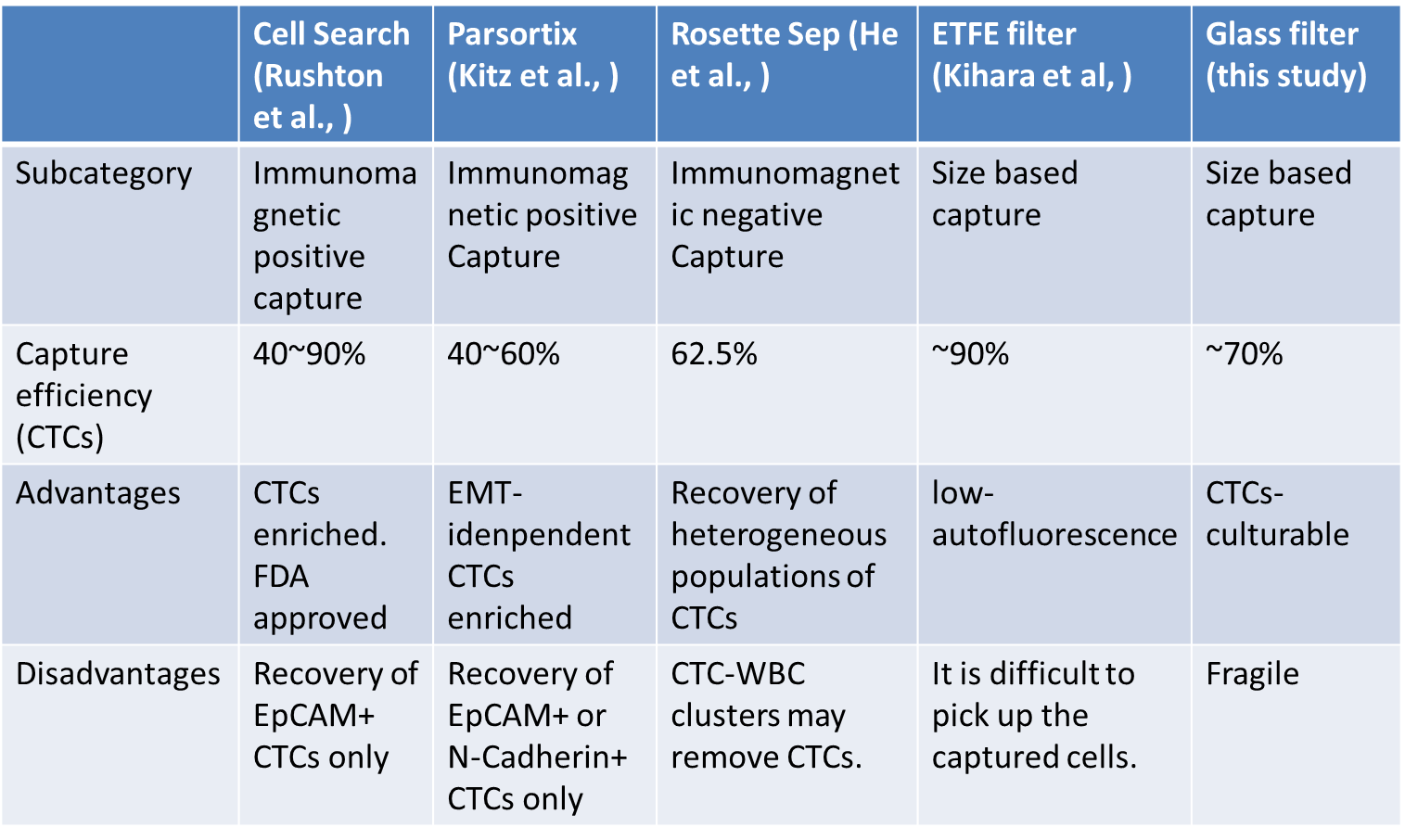
**

**Supplemental Table 1: A comparison of methods to capture CTCs**
